# The relationship between body composition and blood pressure among primary school children in Eastern Cape province, South Africa

**DOI:** 10.4102/phcfm.v11i1.2000

**Published:** 2019-10-02

**Authors:** Howard Gomwe, Eunice Seekoe, Philemon Lyoka, Chioneso S. Marange

**Affiliations:** 1 Faculty of Health Sciences, University of Fort Hare, East London, South Africa; 2 Faculty of Science and Agriculture, University of Fort Hare, East London, South Africa

**Keywords:** blood pressure, body mass index, children, hypertension, South Africa

## Abstract

**Background:**

In South Africa, especially in the Eastern Cape province, despite reported high prevalence of underweight and obesity, little is known regarding the relationship of body composition (BC) with blood pressure (BP) in primary school children. Understanding the relationship between BC and BP in these children is important because it is associated with adverse effects on health and social repercussion in both adolescence and adulthood.

**Aim:**

The aim of this study was to examine the relationship between BC and BP among South African primary school children.

**Setting:**

This study was conducted on a cohort of primary school learners in the Eastern Cape Province of South Africa.

**Methods:**

A school-based cross-sectional survey was conducted among 876 school children aged 9–14 years, using multistage sampling techniques. Body mass and stature were measured using a calibrated scale. Anthropometric measurements including weight, height, waist circumference, triceps, gluteal and subscapular were also collected. Body mass index, percentage body fat and waist-to-hip ratio were calculated.

**Results:**

Of the 876 participants, 356 (40.6%) were boys and 520 (59.0%) were girls. The Spearman’s rho correlation coefficients revealed positive significant correlations between systolic BP with age (*r* = 0.171; *p* < 0.0001), stature (*r* = 0.205; *p* < 0.0001), weight (*r* = 0.277; *p* < 0.0001), body mass index (*r* = 0.243; *p* < 0.0001), waist circumference (*r* = 0.259; *p* < 0.0001), gluteal (*r* = 0.214; *p* < 0.0001), triceps (*r* = 0.203; *p* < 0.0001), subscapular (*r* = 0.167; *p* < 0.0001), body fat percentage (*r* = 0.206; *p* < 0.0001), fat mass (*r* = 0.257; *p* < 0.0001) and fat-free mass (*r* = 0.238; *p* < 0.0001). There was no statistically significant correlation between waist-to-hip ratio and systolic BP (*r* = 0.064; *p* = 0.059). In terms of diastolic BP, there existed no significant correlations with age (*r* = 0.026; *p* = 0.443) and waist-to-hip ratio (*r* = 0.002; *p* = 0.947). Collectively, the prevalence of hypertension was 76.4% in the normal group compared with those who were pre-hypertensive (18.4%) and hypertensive (5.3%). Girls showed a higher prevalence of pre-hypertension than boys (19.6% compared with 16.6%, respectively).

**Conclusion:**

There is a relationship between most of the BC variables and BP in children. The screening of BP as part of physical examinations of school children is necessary for early prevention and intervention programmes for hypertension.

## Introduction

Childhood obesity and overweight is currently one of the major challenges among school children in most parts of South Africa.^1^ Overweight is defined as excessive or abnormal fat accumulation that may impair health. If the body mass index (BMI) is between 25 and 29.9, one is considered overweight. Underweight is considered when BMI is 0–18.5; normal weight is considered when BMI is 18.5–25.0 and obese is considered when BMI is 30.0. Fat is associated with a number of health problems, such as cardiovascular disease and metabolic, pulmonary, neurological, orthopaedic and social disorders.^2^ Increased weight is associated with an increase in hypertension rates, which could lead to atherosclerotic disease later in life.^2,3^ Several studies on the prevalence of high blood pressure (HBP) among children have been conducted in developed countries.^3,4^ However, few studies have been conducted in developing countries.

According to the World Health Organization (WHO) report,^5^ it is estimated that there are 250 million obese children in the world. The prevalence of obesity and overweight is classified as the fifth leading cause of global mortality.^6^ It is also one of the leading health challenges resulting specifically in chronic diseases such as hypertension, metabolic abnormalities, heart disease, diabetes and psychosocial problems in the 21st century.^7^ High blood pressure (BP) during childhood increases cardiovascular risk, such as atherosclerosis, in later adulthood.^8^ Therefore, controlling HBP should start from childhood. The factors that influence BP in children include age, sex and body composition (BC).^9,10^ To understand the relationship between BP and overweight in small children, the following factors must be analysed first: age, sex and BC.

Anthropometric measurements are a non-invasive and inexpensive method to assess children’s nutritional status and have been suggested for wide use in clinical practice. Measuring abdominal fat in addition to obesity has been widely used to improve cardio-metabolic risk assessment^11,12^ because the pattern of fat distribution strongly influences cardio-metabolic risk,^13^ and as a result, abdominal obesity seems to predict the development of cardiovascular diseases better than overall obesity.^14^ The waist circumference (WC) and the waist-to-hip ratio (WHR), which is determined by dividing the WC by hip circumference (HC), have become widely accepted measures for assessing abdominal obesity.^15,16^

According to the evaluation of the relationship between BC and BP measurements of children from rural areas in South Africa, the prevalence of hypertension was evident from school children from 10 to 13 years for girls and boys.^17^ The prevalence of hypertension observed was 4.8% in 17 boys and 5.1% in 29 girls. Elevated BP was confirmed on repeated visits before characterising children as having hypertension. The study provided important information on hypertension among rural South African children.

However, abdominal obesity and anthropometric measurements are more prone to mistakes than BMI measurements, even when they are performed by well-trained specialists.^18^ Body mass index is the most common and reliable measure of overall obesity.^19^ Little is known about the relationship between BC and BP in children in the Eastern Cape Province. The aim of this study was to evaluate the relationship between BC and BP in primary school children.

## Subjects and methods

### Design

The researcher used a cross-sectional descriptive study design for data collection in which BCs and BP were measured among the children. The study involved a random sample of 876 learners aged 9–14 years.

### Sampling

The project involved three randomly selected district municipalities in the Eastern Cape Province, South Africa. Two randomly selected educational districts from each municipality were then used to randomly select the schools that were used to draw the sample. The District Education Department of each selected municipality provided a list of schools on which the random selection was based. Overall, 18 primary schools from quintiles 1, 2 and 3 in each district were randomly selected using a computer-generated programme. Class registers were then used to randomly draw the sample from the randomly selected schools.

### Anthropometric measurements

Body mass, stature and skinfolds were measured using the protocol of the International Society for the Advancement of Kinanthropometry.^20^ Body composition indices including BMI (weight/height^2^) were calculated.^21^ The sums of two skinfolds (triceps and subscapular) were calculated and^22^ an equation was used to predict percentage body fat in school children. Waist-to-hip ratio is an anthropometric measurement used for predicting abdominal fat and the related risk of cardiovascular disease. To determine WHR, WC was measured in centimetres and subsequently divided by measurements of HC according to the formula by Lee and Nieman.^23^

Waist circumference was measured according to standardised procedures described by Hammond and Gibson.^24^ A tape measure was used to measure WC. The measurements in centimetres (cm) were taken at the narrowest area below the rib cage and above the umbilical cord as viewed from the front. The measurements were taken at the end of a normal expiration. These techniques are standardised, which contributes to the consistency and reliability of the calculated WHR values.

Hip gluteal was measured to the nearest 0.1 cm, according to standardised methods by Hammond and Gibson.^24^ A tape measure was used and was placed in a horizontal plane around the hips at the point of greatest circumference. The measurement was taken in close contact with the skin, but without indenting the soft skin tissue of the participant. The measurements were recorded in cm.

### Blood pressure

Blood pressure was measured three times on the left upper arm at 5-min interval using an electronic BP monitor (Omron HEM – 705 CP Device, Tokyo, Japan), according to standardised guidelines (National Heart, Lung, and Blood Institute/National High Blood Pressure Education Programme [NHBPEP] Coordinating Committee).^25^ The children had to relax first before the measurement for about 5 min. The children were in a seated position using age-appropriate child BP cuffs. The procedure was explained to the participants before the measurement and the cuff inflated and deflated once. Blood pressure measurements were categorised according to systolic blood pressure (SBP) and/or diastolic blood pressure (DBP) percentiles as follows: normal BP – average SBP and/or average DBP < 90th percentile; pre-hypertension – average SBP and/or average DBP ≥ 90th percentile but < 95th percentile; and hypertension – average SBP and/or average DBP ≥ 95th percentile.^26^ The average of the three BP measurements was recorded and included in the statistical analysis.

### Pilot testing

A pilot test was conducted before the actual study to confirm that the tools were able to yield the intended result. Four research assistants from Human Movement Sciences and two qualified nurses underwent 1-week training course in body measurements. The research assistants had a special training in anthropometry level 1 from the International Society for the Advancement of Kinanthropometry (ISAK).

## Statistical analysis

Data were analysed using descriptive statistics, such as means and standard deviations. A non-parametric Mann–Whitney *U* test was used to assess gender differences between anthropometric and physiological characteristics of school learners. To study the relationship among the variables, Spearman’s rho correlation coefficients were computed. The statistical analysis was done using the Statistical Package for Social Sciences (version 24).

## Ethical considerations

All ethical issues including informed consent, plagiarism, misconduct, data fabrication and/or falsification, double publication and/or submission, and so on have been completely observed by the authors.

## Results

Of the 876 participants, 356 (40.6%) were boys and 520 (59.0%) were girls. Table 1 depicts the anthropometric and physiological characteristics of the school learners. The mean age of learners was 11.0 ± 1.5 years, with boys having a significantly higher mean age (11.2 ± 1.5 years) compared with that of girls (10.9 ± 1.4 years). The mean weight was 39.3 kg ± 10.35 kg. The mean height of the learners was 144.0 cm ± 10.94 cm, with no significant differences in height between boys (144.2 cm ± 10.43 cm) and girls (143.9 cm ± 11.08 cm). Boys, on average, had a significantly lower WC (75.7 cm ± 8.48 cm) and also presented with a lower WHR (1.2 cm ± 0.16 cm) than the girls (79.1 cm ± 10.51 cm and 1.2 cm ± 0.30 cm, respectively). The mean of gluteal, triceps and subscapular was significantly higher for girls (65.0 cm ± 9.12 cm; 14.2 mm ± 6.09 mm and 10.5 mm ± 6.54 mm, respectively) than for the boys (63.5 cm ± 9.07 cm; 10.3 mm ± 5.51 mm and 7.0 mm ± 4.23 mm, respectively). The sample reported a mean fat mass percentage of 20.0% (±7.35), while the overall mean fat mass was 8.4 kg (±4.97) and a mean fat-free mass of 31.0 kg ± 6.52 kg. In all these cases, the girls had significantly higher mean levels for mean fat mass percentage (mean = 22.3%; standard deviation [s.d.] = 6.73; *z* = -12144; *p* < 0.0001), mean fat mass (mean = 9.5 kg; s.d. = 5.06; *z* = -9.725; *p* < 0.0001) and mean fat-free mass (mean = 30.9 kg; s.d. = 6.98; *z* = 1.375; *p* < 0.0001). Concerning the BP evaluation, statistically significant differences between boys and girls were only found in DBP (*z* = -2.774; *p* = 0.006), with the boys having a significantly lower DBP (65.3 mmHg ± 13.53 mmHg) compared with that of girls (67.6 mmHg ± 13.07 mmHg).

**TABLE 1:** Anthropometric and physiological characteristics of school learners.

Variables	Combined (*n* = 876)	Boys (*n* = 356)	Girls (*n* = 520)	Mann–Whitney *U Z*-test statistic	*p*
	Mean ± s.d.	Mean ± s.d.	Mean ± s.d.		
Age (years)	11.0 ± 1.5	11.2 ± 1.5	10.9 ± 1.4	−3.184	0.001*
Weight (kg)	39.3 ± 10.35	37.9 ± 8.58	40.3 ± 11.31	−2.717	0.007*
Height (cm)	144.0 ± 10.94	144.2 ± 10.43	143.9 ± 11.08	−0.368	0.713
BMI (kg/m^2^)	18.9 ± 4.26	18.1 ± 3.02	19.3 ± 4.65	−3.827	0.0001*
WC (cm)	77.7 ± 9.87	75.7 ± 8.48	79.1 ± 10.51	−5.018	< 0.0001*
WHR (cm)	1.2 ± 0.26	1.2 ± 0.16	1.2 ± 0.30	−2.528	0.012*
Gluteal (cm)	64.4 ± 9.12	63.5 ± 9.07	65.0 ± 9.12	−3.229	0.001*
Triceps (mm)	12.6 ± 6.16	10.3 ± 5.51	14.2 ± 6.09	−11.172	< 0.0001*
Subscapular (mm)	9.1 ± 5.97	7.0 ± 4.23	10.5 ± 6.54	−11.727	< 0.0001*
FATp (%)	20.0 ± 7.35	16.8 ± 6.96	22.3 ± 6.73	−12.144	< 0.0001*
FAT mass (kg)	8.4 ± 4.97	6.7 ± 4.33	9.5 ± 5.06	−9.725	< 0.0001*
FAT-free mass (kg)	31.0 ± 6.52	31.2 ± 5.81	30.9 ± 6.98	1.375	0.169
SBP (mmHg)	107.9 ± 19.42	106.8 ± 19.12	108.6 ± 19.60	−1.023	0.306
DBP (mmHg)	66.7 ± 13.29	65.3 ± 13.53	67.6 ± 13.07	−2.774	0.006*

s.d., standard deviation; BMI, body mass index; WC, waist circumference; WHR, waist-to-hip ratio; FATp, body fat percentage; FAT mass, body fat mass; FAT-free mass, body fat-free mass; SBP, systolic blood pressure; DBP, diastolic blood pressure.

* , Statistically significant differences at alpha = 0.05.

Table 2 displays the descriptive profile of the various classifications of SBP and DBP with sex. In terms of classifications by SBP, a greater percentage of participants were in the normal group (76.4%) compared with the pre-hypertension (18.4%) and hypertension (5.3%) groups. Girls showed a higher prevalence of pre-hypertension than the boys (19.6% compared with 16.6%). Also using SBP, the prevalence of hypertension in girls (5.6%) was higher than that of boys (4.8%). When classifications are done using DBP, the findings were similar to those classified using SBP. Pre-hypertension was detected in 129 (14.7%) and hypertension in 23 (2.6%) of learners. Four hundred and nineteen (80.7%) of the girls had normal BP, 87 (16.7%) had pre-hypertension and 14 (2.7%) had hypertension. On the contrary, 305 (85.7%) of the boys had normal BP, 42 (11.8%) had pre-hypertension and 9 (2.5%) had hypertension.

**TABLE 2:** Descriptive statistics of systolic and diastolic blood pressure with gender.

Blood pressure	Combined (*n* = 876)		Boys (*n* = 356)		Girls (*n* = 520)	
	Frequency	%	Frequency	%	Frequency	%
**SBP (mmHg)**						
Normal	669	76.4	280	78.7	389	74.8
Pre-hypertension	161	18.4	59	16.6	102	19.6
Hypertension	46	5.3	17	4.8	29	5.6
**DBP (mmHg)**						
Normal	724	82.6	305	85.7	419	80.6
Pre-hypertension	129	14.7	42	11.8	87	16.7
Hypertension	23	2.6	9	2.5	14	2.7

SBP, systolic blood pressure; DBP, diastolic blood pressure.

Table 3 shows the non-parametric Spearman’s rho correlation coefficients that were used to determine the relationship of SBP and DBP with the anthropometric and physiological characteristics of the participants. All the correlations were statistically and practically significant for SBP with the anthropometric and physiological characteristics except for WHR (*r* = 0.064; *p* = 0.059). In terms of DBP, there existed no significant correlations with age for all the given categories, that is, combined (*r* = 0.026; *p* = 0.443), boys (*r* = 0.025; *p* = 0.644) and girls (*r* = 0.044; *p* = 0.313). Only triceps (*r* = 0.151; *p* = 0.004), body fat percentage (*r* = 0.133; *p* = 0.012) and body fat mass (*r* = 0.131; *p* = 0.014) had positive significant correlations with DBP for the boys. On the contrary, DBP had significant positive correlations only with weight (*r* = 0.096; *p* = 0.028), BMI (*r* = 0.096; *p* = 0.029), gluteal (*r* = 0.107; *p* = 0.015), triceps (*r* = 0.116; *p* = 0.008), body fat percentage (*r* = 0.094; *p* = 0.032) and body fat mass (*r* = 0.105; *p* = 0.017) for the girls.

**TABLE 3:** Relationships of systolic and diastolic blood pressure with study’s theoretical variables.

Variables	Combined		Boys		Girls	
	SBP (mmHg)	DBP (mmHg)	SBP (mmHg)	DBP (mmHg)	SBP (mmHg)	DBP (mmHg)
	*r* (*p*)	*r* (*p*)	*r* (*p*)	*r* (*p*)	*r* (*p*)	*r* (*p*)
Age (years)	0.171** (0.000)	**0.026** **(0.443)**	0.171** (0.001)	**0.025** **(0.644)**	0.178** (0.000)	**0.044** **(0.313)**
Weight (kg)	0.277** (0.000)	0.103** (0.002)	0.295** (0.000)	**0.098** **(0.065)**	0.265** (0.000)	0.096* (0.028)
Height (cm)	0.205** (0.000)	0.083* (0.014)	0.229** (0.000)	**0.096** **(0.072)**	0.195** (0.000)	**0.079** **(0.072)**
BMI (kg/m^2^)	0.243** (0.000)	0.090** (0.008)	0.208** (0.000)	**0.042** **(0.431)**	0.253** (0.000)	0.096* (0.029)
WC (cm)	0.259** (0.000)	0.094** (0.006)	0.261** (0.000)	**0.081** **(0.128)**	0.256** (0.000)	**0.079** **(0.073)**
WHR (cm)	**0.064** **(0.059)**	**0.002** **(0.947)**	**0.099** **(0.061)**	**0.005** **(0.932)**	**0.033** **(0.457)**	**0.014** **(0.757)**
Gluteal (cm)	0.214** (0.000)	0.098** (0.004)	0.167** (0.002)	**0.062** **(0.247)**	0.236** (0.000)	0.107* (0.015)
Triceps (mm)	0.203** (0.000)	0.156** (0.000)	0.206** (0.000)	0.151** (0.004)	0.209** (0.000)	0.116** (0.008)
Subscapular (mm)	0.167** (0.000)	0.095** (0.005)	0.165** (0.002)	**0.083** **(0.117)**	0.183** (0.000)	**0.050** **(0.251)**
FATp (%)	0.206** (0.000)	0.136** (0.000)	0.210** (0.000)	0.133* (0.012)	0.220** (0.000)	0.094* (0.032)
FAT Mass (kg)	0.257** (0.000)	0.136** (0.000)	0.275** (0.000)	0.131* (0.014)	0.265** (0.000)	0.105* (0.017)
FAT-Free Mass (kg)	0.238** (0.000)	0.070* (0.037)	0.256** (0.000)	**0.060** **(0.258)**	0.233** (0.000)	**0.084** **(0.057)**

Note: Bold data represent non-significant correlations.

* , Correlation is remarkable when significant level is 0.05 (one-tailed test).

** , Correlation is remarkable when significant level is 0.01(one-tailed test).

## Discussion

This study examined the relationship between BC and BP among school children in the Eastern Cape Province. Although the relationship between BC and BP has been extensively studied, to our knowledge, this is the first study to examine the relationship in the Eastern Cape Province. The findings demonstrate age and sex differences in the BP of children. The study showed several anthropometric variables that were significantly correlated with SBP in the children, including stature, weight, BMI, WC, triceps, subscapular, gluteal skinfold thicknesses and percentage body fat except WHR. All anthropometric measurements were statistically higher among children with HBP as compared with those with normal BP. The close relationship between body weight and HBP has been described in children.^27,28,29^

The present findings indicated that body mass and stature in boys and body mass in girls were the most influential predictors of SBP in the East Cape children. The observation that age and body size in boys account for a greater proportion of SBP variations than percentage body fat was consistent with other studies.^17,30^ This suggests that anthropometric measurements other than body fat may be age and body size that account for a greater proportion of SBP.

The results showed that overweight children had significantly higher SBP and DBP than the non-obese, confirming previous observations that greater BMI in children is associated with raised BP.^27^ In addition, the BP status of the children was defined according to the recommendations of the WHO Expert Committee on Hypertension Control,^31^ which were based on the sex- and age-specific guidelines of the Second Task Force of Blood Pressure Control in Children.^31^ Using the 85th and 95th sex- and age-specific BMI cut-off points, it was found that obese children have a substantially greater risk of having HBP; obese boys and girls were 3·8 and 11 times more likely to have HBP than the underweight, respectively. In the present study, the risk of having elevated BP also increased with increasing levels of BMI. The risk of having elevated BP also increased with increasing levels of percentage body fat. These results were similar with the results of earlier studies in which increased triceps skinfold thickness^25^ or excess percentage body fat^7^ was used to examine the relationship between BP and adiposity in children and adolescents. However, these data indicate the role of HBP and overweight at this age in increasing the risk of cardiovascular disease in adulthood.^32,33^ Researchers found that being overweight in children and adolescents was associated with an increased risk of morbidity from congenital heart disease and atherosclerosis in adulthood. The effects of overweight in children on adult morbidity and mortality may be related to the accumulation of fat that occurs during the time of puberty. Studies on children and adolescents showed that increased body fatness was significantly associated with a variety of cardiovascular and metabolic abnormalities that cluster with obesity, including elevated BP and lipoprotein profile disturbances.^34^

## Limitations of the study

The limitation of this study is that the BP reading was recorded as the average of three measurements, which were taken after 5-min rest, but on one occasion only. Thus, the possibility that errors may have occurred in classifying children as having HBP or normal BP cannot be ruled out. However, the purpose of using BP categories in the analysis was to obtain a general idea of the extent of elevated BP in the studied school children rather than to diagnose the presence of hypertension among them.

## Conclusion and recommendations

Prevalence of body weight disorders and pre-hypertension was observed in this research study. There were positive significant correlations between SBP and age. There was no statistically significant correlation between WHR and SBP. In terms of DBP, there existed no significant correlations with age and WHR. We demonstrated that there is a positive relationship between BC and BP among primary school children. The fact that HBP often starts from childhood to adulthood makes childhood a crucial age to investigate BC and its association with hypertension. These data can provide important insights to develop appropriate strategies and suitable interventions to curb associated risk factors and optimise health lifestyle.
